# Biomechanical evaluation of autologous bone-cage in posterior lumbar interbody fusion: a finite element analysis

**DOI:** 10.1186/s12891-020-03411-1

**Published:** 2020-06-13

**Authors:** Haodong Zhu, Weibin Zhong, Ping Zhang, Xiaoming Liu, Junming Huang, Fatai Liu, Jian Li

**Affiliations:** 1 Department of Orthopaedic Surgery, The Fifth Affiliated Hospital of Guangzhou Medical University, Guangzhou, 510700 China; 2 Department of Orthopaedic Surgery, The Third Affiliated Hospital of Guangzhou Medical University, Guangzhou, 510150 China

**Keywords:** Biomechanical evaluation, Autologous bone-cage, Posterior lumbar interbody fusion, Finite element analysis

## Abstract

**Background:**

An autologous bone-cage made from the spinous process and laminae might provide a stability in posterior lumbar interbody fusion (PLIF) close that of the traditional-cage made of polyetheretherketone (PEEK) or titanium. The biomechanical effect of autologous bone-cages on cage stability, stress, and strains, and on the facet contact force has not been fully described. This study aimed to verify whether autologous bone-cages can achieve similar performance as that of PEEK cages in PLIF by using a finite element analysis.

**Methods:**

The finite element models of PLIF with an autologous bone-cage, a titanium cage, and a PEEK cage were constructed. The autologous bone-cage was compared with the titanium and PEEK cages. The mechanical properties of the autologous bone-cage were obtained through mechanical tests. The four motion modes were simulated. The range of motion (ROM), the stress in the cage-end plate interface, and the facet joint force (FJF) were compared.

**Results:**

The ROM was increased at adjacent levels but decreased over 97% at the treated levels, and the intradiscal pressure at adjacent levels was increased under all conditions in all models. The FJF disappeared at treated levels and increased under extension, lateral bending, and lateral rotation in all models. The maximum stress of the cage-endplate interface was much lower in the autologous bone-cage model than those in the PEEK and titanium cage models.

**Conclusions:**

In a finite model of PLIF, the autologous bone-cage model could achieve stability close that of traditional titanium or PEEK cages, reducing the risk of subsidence.

## Background

Posterior lumbar interbody fusion (PLIF) is widely used in the treatment of lumbar conditions like degenerative disc disease, spondylolisthesis, and trauma. The use of cages is vital to restore disc height, correct coronal and sagittal alignment, and achieve indirect decompression [1, 2]. The placement of interbody cages through a posterior approach might decrease the range of motion (ROM) and improve stability after the surgery [3, 4]. In addition, the interbody cage used in PLIF improves the loading capacity of the treated levels [3, 4]. Usually, a posterior supplemental fixation is used in PLIF to improve multiplanar stability and load sharing [5, 6].

Traditional cages made of polyetheretherketone (PEEK) or titanium are widely used in lumbar fusion due to their good mechanical properties, which are close to that of cortical bone [2, 7]. Nevertheless, PEEK is a bioinert material, and the use of PEEK cages in lumbar fusion might lead to nonunion, osteolysis, and subsidence [2, 7]. In addition, titanium cages with high mechanical stiffness might improve the risk of subsidence after the surgery [1–7].

**Fig. 1:**
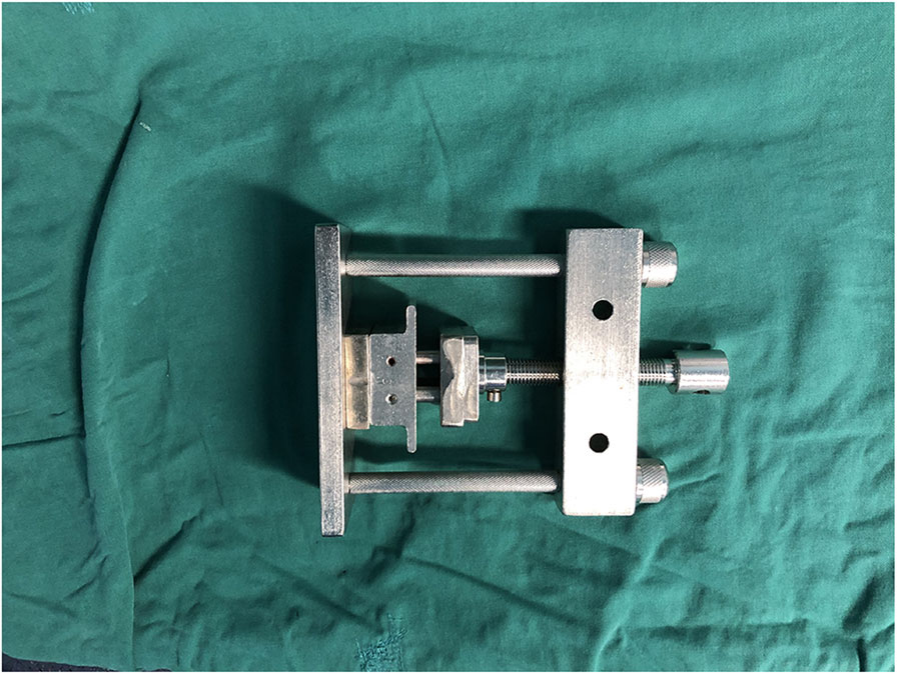
Autologous bone cage forming device

**Fig. 2:**
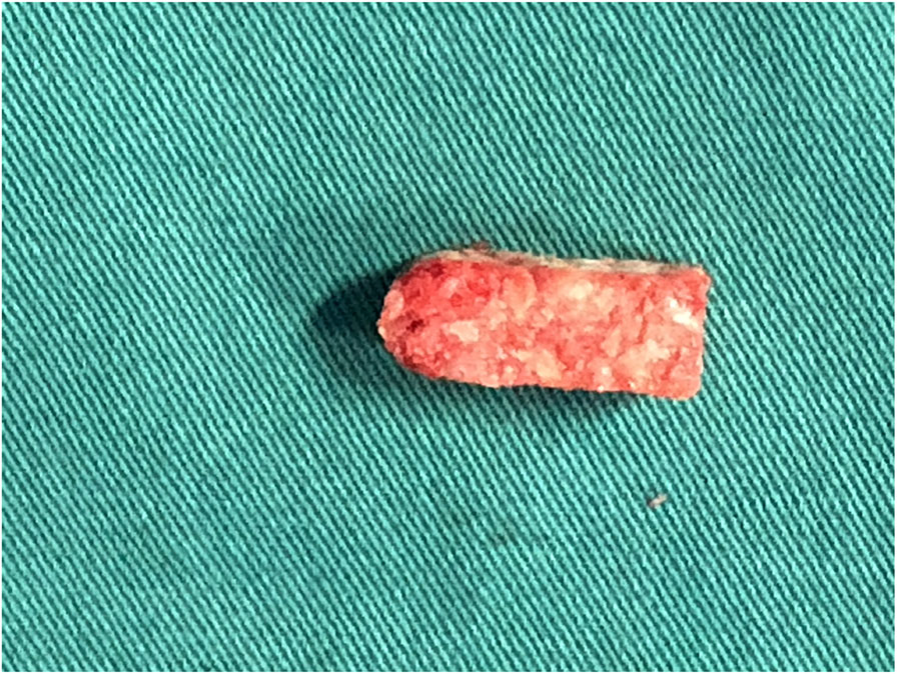
The autologous bone cage used in clinical practice

Previous studies showed a high fusion rate after PLIF using cages made of autologous bone graft from spinous processes and laminae [8]. Recently, clinical and in vitro studies were conducted to examine the biomechanical performance of autologous bone-cages. The biomechanical stability, fusion rate, and safety of PLIF could be maintained using autologous bone-cages [6, 9]. A forming device (China Patent No. ZL201120261348.8) (Fig. 1) has recently been designed for making autologous bone-cages (Fig. 2) using the spinous process and laminae dissected during the surgical procedure. In one of our previous studies, the autologous bone-cage showed good performance in biomechanical in vitro experiments [9].

Although some parameters can be studied through in vitro biomechanical tests or clinical studies, some of them cannot be measured directly [6, 9, 10]. Finite element analysis (FEA) has been widely used for the evaluation of the biomechanical behaviors of different cages during lumbar fusion [10–12].

Therefore, using the FEA model to study the biomechanical effects of autologous bone-cages could provide valuable information, and stress and strains on autologous bone-cages, facet joint force, and ROM could be determined. This study aimed to evaluate the biomechanical performance of autologous bone-cages by comparing them with traditional PEEK and titanium cages using FEA.

## Methods

### Images data acquisition

Computed tomography (CT) images of an intact lumbar spine at L3-S1 were obtained from a 25-year-old male volunteer, without any lumbar diseases, as confirmed by physical and radiographic examination. The data were exported in the DICOM format.

### Reconstruction

The images were imported into the Mimics software (Materialise Inc., Leuven, Belgium). The 3D geometric structures of L3-S1, including the vertebral body and the intervertebral disc, were reconstructed. The 3D models of L3-S1 were exported as binary STL point cloud data. The STL data files of L3-S1 were then imported to Geomagic Studio 2013 (3D Systems, Ltd., USA) to de-feature and smoothen the surface of the vertebral body. Nonuniform rational b-spline surfaces (NURBS) were finally created, and the model was exported as an IGS file.

### Cage and fixator modeling

**Fig. 3:**
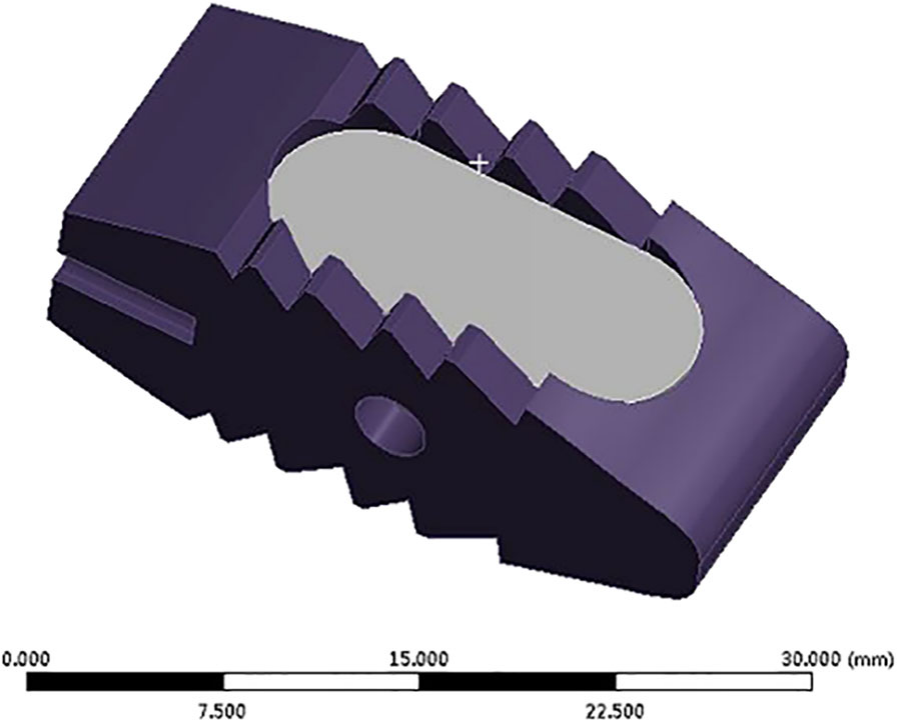
Model of PEEK/titanium cage with bone graft

**Fig. 4:**
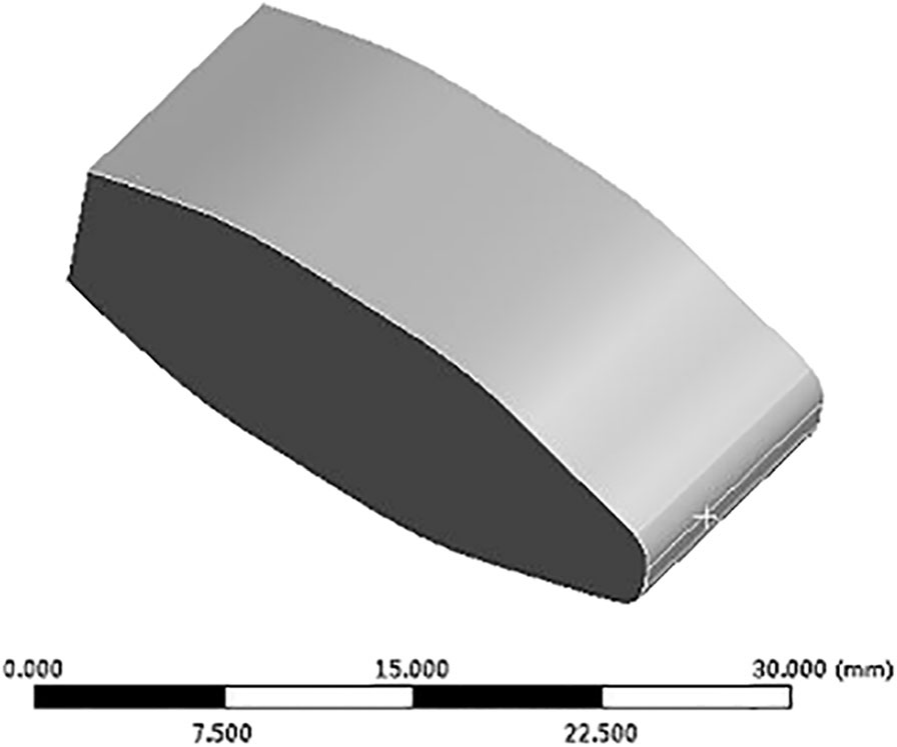
Model of the autologous bone cage

**Fig. 5:**
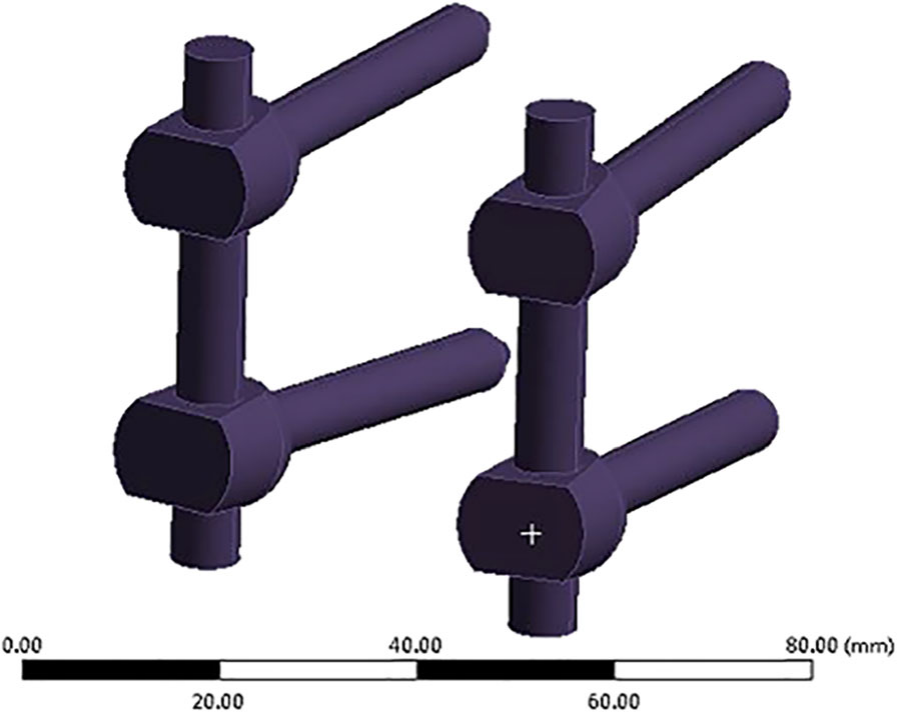
Model of posterior instruments

A traditional cage and autologous bone-cage used for PLIF was constructed in Solidworks 2014 (Dassault Inc., Concord, USA) (Figs. 3 and 4) and exported as an STL file. The screws and rods were built with simplified geometric structures in Solidworks and exported as STL files as well (Fig. 5).

### FEA model establishment

**Fig. 6:**
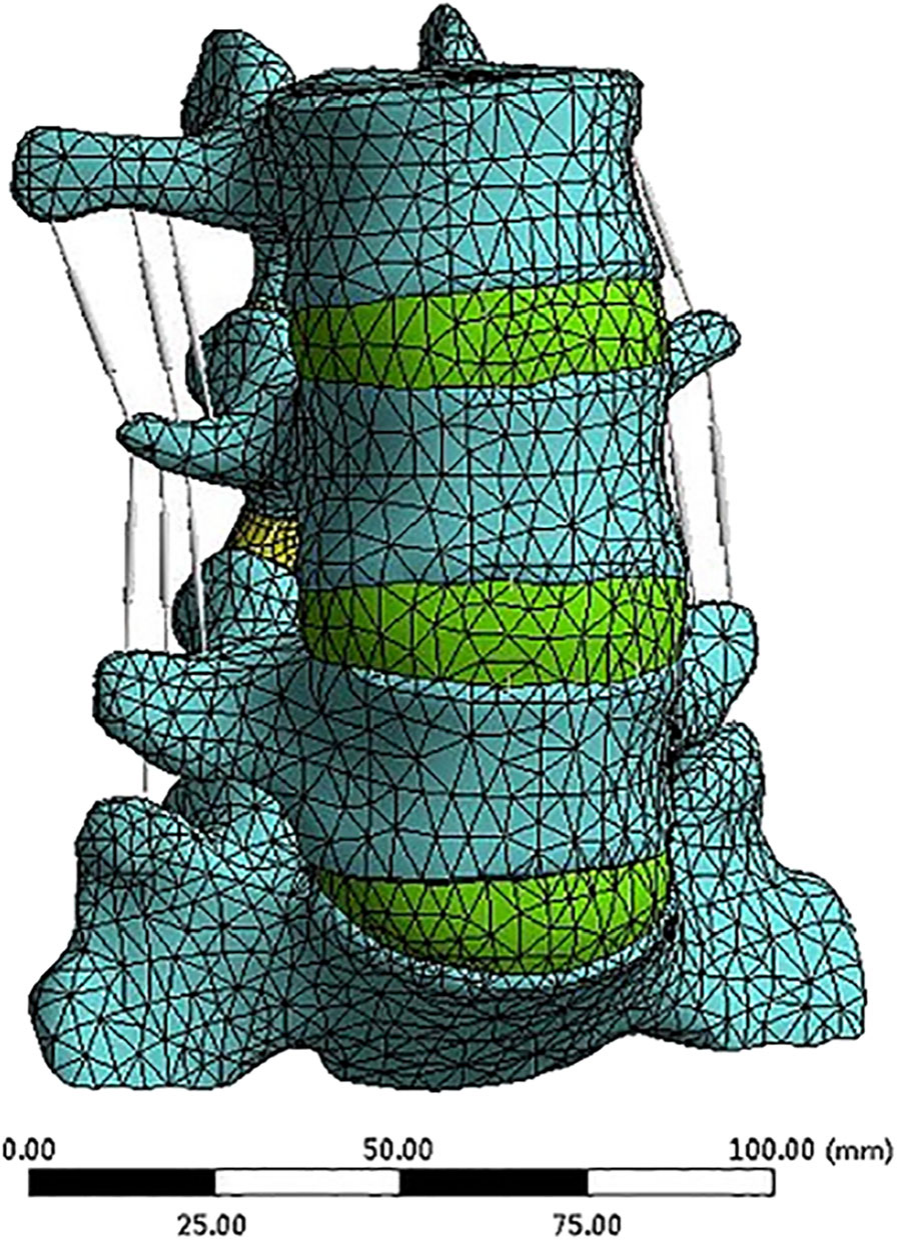
Intact model of L3-S

The geometric structures were meshed in ANSYS Workbench (ANSYS, Inc., Southpointe, USA). Each vertebral body was divided into three parts: cortical bone, cancellous bone, and endplate. Each intervertebral disc was divided into the annulus ground and nucleus pulposus. The mesh quality assessment was made according to the literature of Burkhart et al. [13]. The cartilages were meshed with hexahedral elements and the other parts with the tetrahedral elements (Fig. 6). The intact model of L3-S1 was made up of 28,288 elements and 55,559 nodes (Table 1). Seven spinal ligaments, including the anterior longitudinal ligament, the posterior longitudinal ligament, the supraspinous ligament, the interspinous ligament, the transverse ligament, the ligamentum flavum, and the capsular ligament, were modeled with two-node truss elements [6]. The material properties were assigned according to the literature (Table 2) [6, 10–14]. The cortical bone and the endplate were 1.0-mm and 0.5-mm thick, respectively [6, 10, 11]. The parameters of all ligaments were obtained from the literature and assigned to be tension-resistant only [14]. The contact between the facet joints was defined as face-to-face contact with a friction coefficient of 0.1.

**Table 1:** Mesh information

	nodes	elements
group1	93,934	46,409
group2	93,934	46,409
group3	91,575	44,884
intact	55,559	28,288

**Table 2:** Material properties of components [5xiao, 10 zhang m 15faizan, 17 Vadapalli S, 18 Lin B, 19zhang World Neurology, 20zhang Biomed Mater, 21Zhang Z Comput Methods Biomech Biomed Engin 23zhong]

Element Set	Young’s modulus (MPa)	Poisson’s Ratio (μ)
Cortical bone	12,000	0.3
Cancellous bone	100	0.2
Anulus	4.2	0.45
Nucleus pulposus	1.0	0.4999
Anterior longitudinal ligament	15	
Posterior longitudinal ligament	10	
Transverse ligament	10	
Interspinous ligament	10	
Supraspinous ligament	8	
Ligamentum flavum	15	
Capsular ligament	7.5	
Cage (PEEK)	3600	0.3
Cage (Titanium)	110,000	0.3
Cage (Autologous bone)	5000	0.29
Pedicle screws (Titanium)	110,000	0.3
Rods (Titanium)	110,000	0.3
Bone graft	1940	0.3

**Fig. 7:**
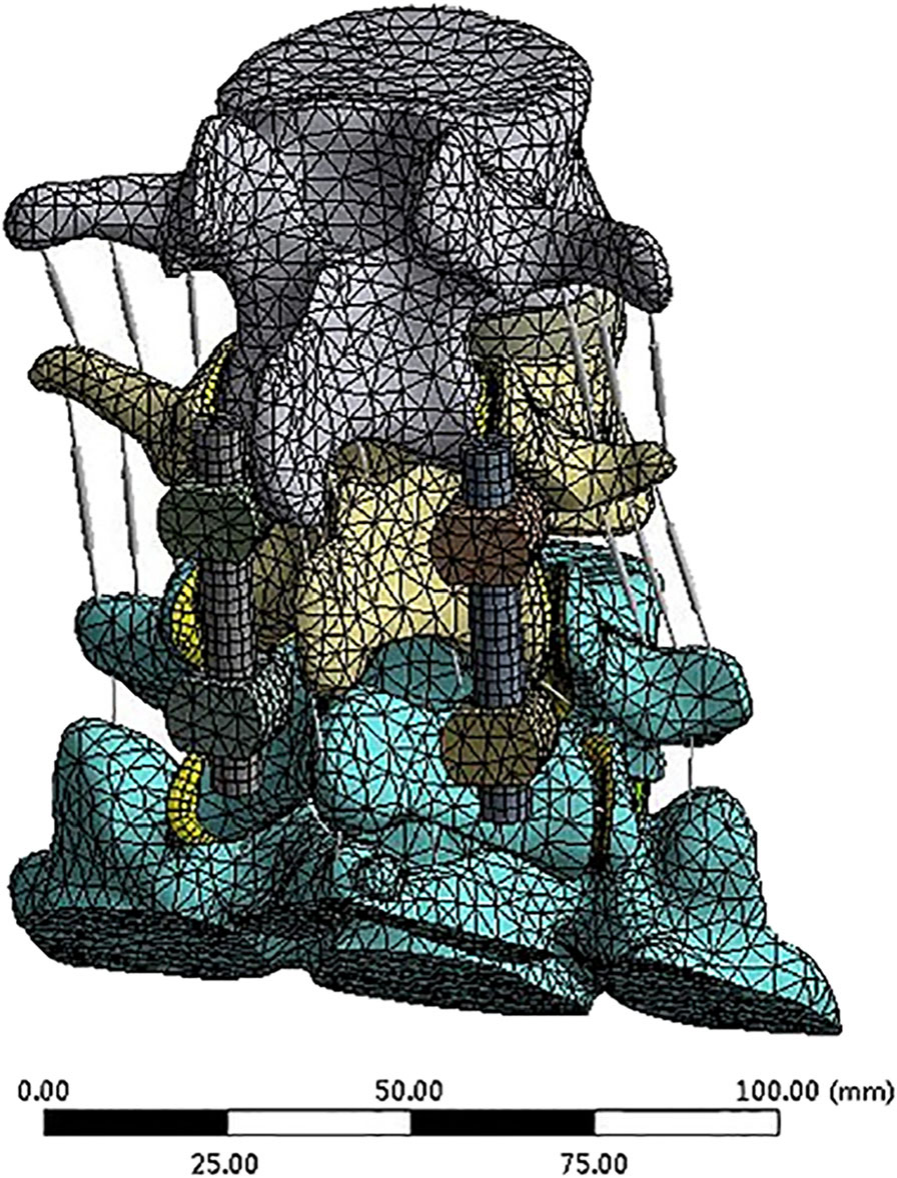
Model of PLIF with posterior instruments

**Fig. 8:**
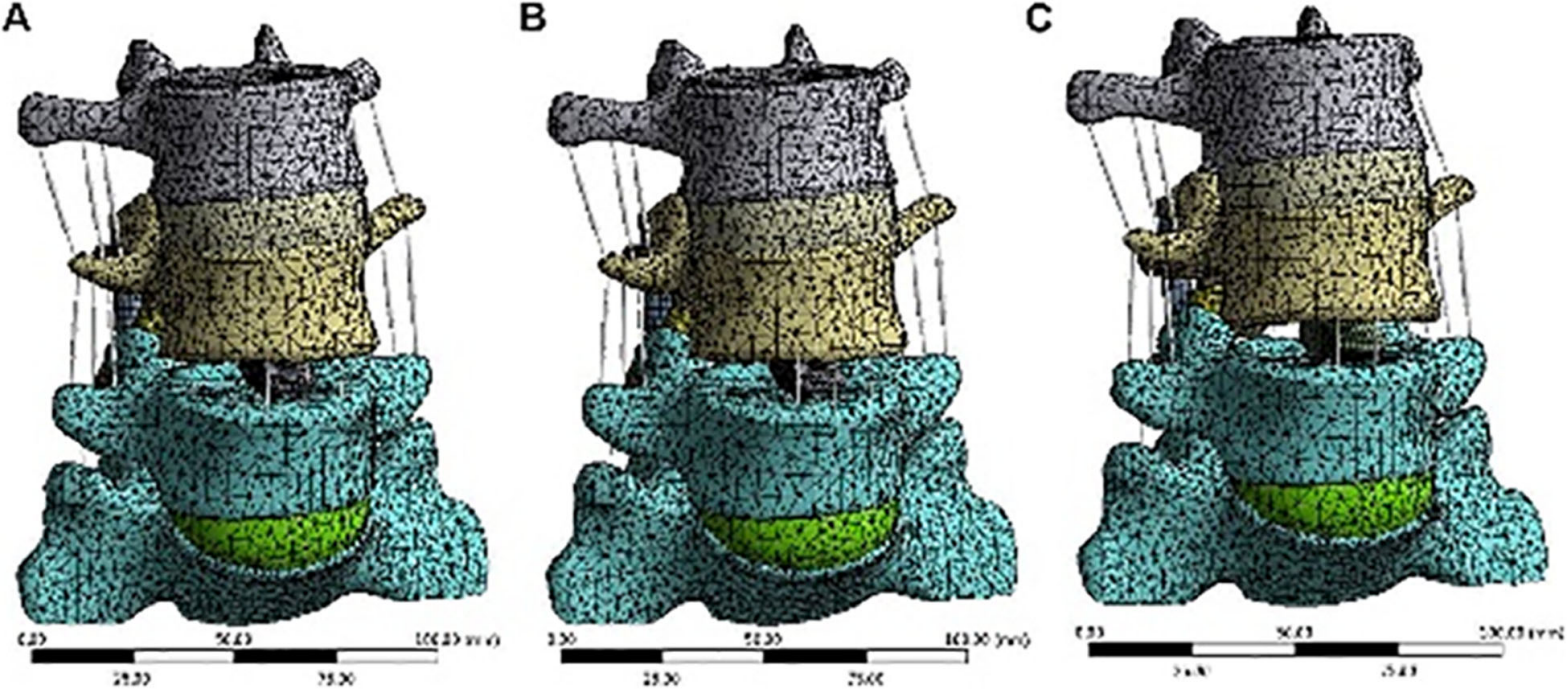
Model of PLIF at the L4-L5 level with titanium/PEEK/autologous bone cage, respectively

To simulate PLIF, the whole intervertebral disc at the L4-L5 level was removed. Four pedicle screws connected by two rods were inserted into the pedicles of L4 and L5 (Fig. 7). The interbody cages made of titanium, PEEK, and autologous bone was placed in the middle of the intervertebral space (Fig. 8). The bone graft was inserted in the titanium cage and PEEK cage model. As the material property of the autologous bone-cage is unclear, the properties were set at a Young’s modulus of 5000 MPa, with a Poisson’s ratio of 0.29. The interactions between cages and endplates were defined as face-to-face contact with a friction coefficient of 0.2. The interaction property “TIE” was defined as the interactions between (1) pedicle screws and pedicles, (2) pedicle screws and vertebral bodies, and (3) pedicle screws and rods. Finally, three models of PLIF simulation were built: (1) PLIF with PEEK cage, (2) PLIF with titanium cage, and (3) PLIF with autologous bone-cage. The number of elements and nodes of the three models are shown in Table 2.

### Boundary and load condition

The bottoms of the models were fixed rigidly. All models were stressed with an axial compressive load of 400 N and 7.5 Nm on the superior surface of the L3 vertebral body to simulate flexion, extension, left bending, and left axial rotation, considering that the models were symmetric on the midsagittal plane [12]. The ROM of L3/4, L4/5, and L5/S1, facet joints force (FJF) of adjacent levels and Von Mise stresses in cage-endplate interfaces, and intradiscal pressure (IDP) of L3/4 and L5/S1 were calculated.

## Results

### Model validation

To validate the model of intact lumbar spine L3-S, the ROM at the L3/4 level was compared to the literature [15]. The ROMs at the L3/4 level in our model were within the reported range [15].

### Range of motion

**Fig. 9:**
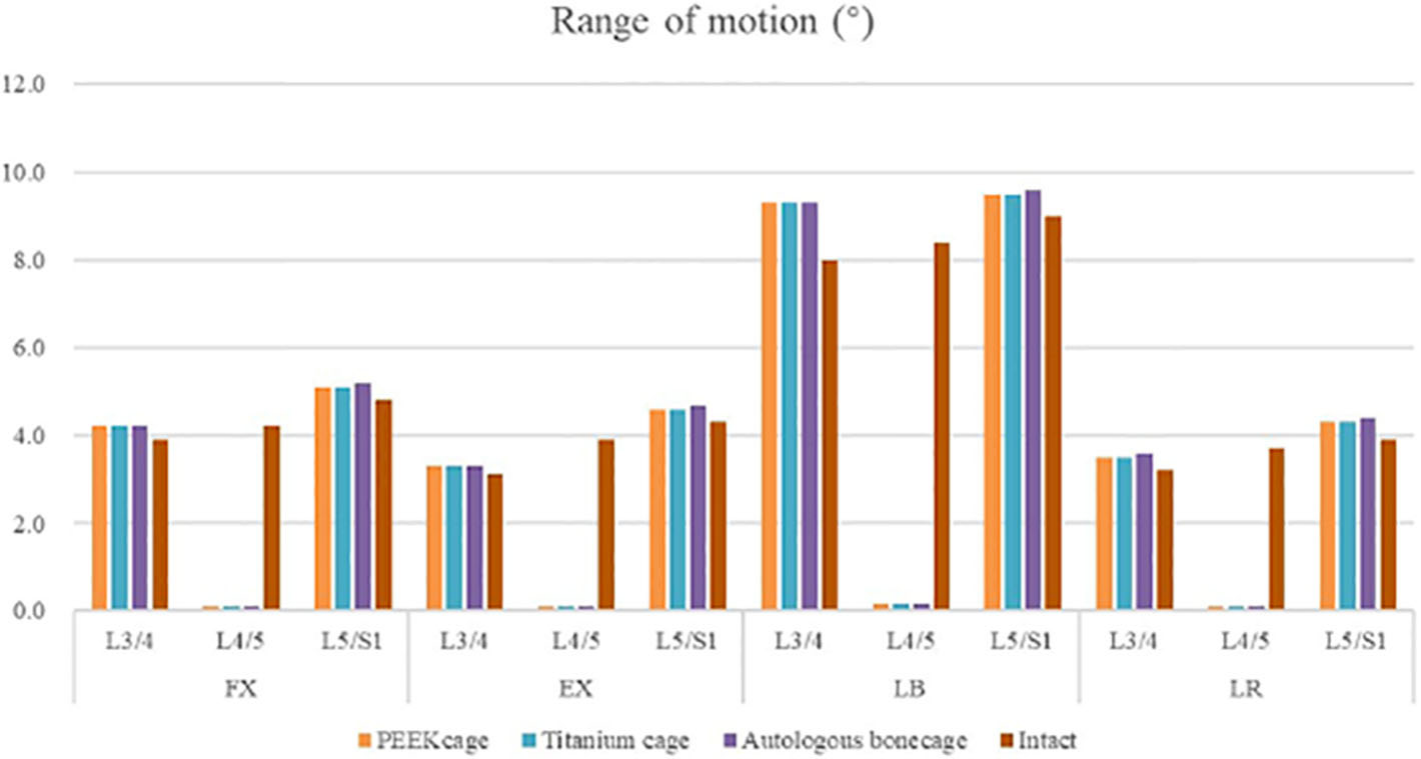
Range of motion at various levels for various cages in 4 motion modes

Under the axial compressive load of a combination of 400 N and 7.5 Nm, the ROM was intact, and the treated models are shown in Fig. 9 and Tables 3 and 4.

**Table 3:** Rang of motion in different motion modes (°)

		PEEK cage	Titanium cage	Autologous bone cage	Intact
FX	L3/4	4.2	4.2	4.2	3.9
	L4/5	0.1	0.1	0.1	4.2
	L5/S	5.1	5.1	5.2	4.8
EX	L3/4	3.3	3.3	3.3	3.1
	L4/5	0.1	0.1	0.1	3.9
	L5/S	4.6	4.6	4.7	4.3
LB	L3/4	9.3	9.3	9.3	8.0
	L4/5	0.2	0.2	0.2	8.4
	L5/S	9.5	9.5	9.6	9.0
LR	L3/4	3.5	3.5	3.6	3.2
	L4/5	0.1	0.1	0.1	3.7
	L5/S	4.3	4.3	4.4	3.9

**Table 4:** Change of range of motion in different motion modes

		PEEKcage	Titanium cage	Autologous bone cage
FX	L3/4	8%	8%	8%
	L4/5	−98%	−98%	−98%
	L5/S1	6%	6%	8%
EX	L3/4	6%	6%	6%
	L4/5	−97%	−97%	−97%
	L5/S1	7%	7%	9%
LB	L3/4	16%	16%	16%
	L4/5	−98%	−98%	−98%
	L5/S1	6%	6%	7%
LR	L3/4	9%	9%	13%
	L4/5	−97%	−97%	−97%
	L5/S1	10%	10%	13%

The ROMs at the L4/5 level were 0.1°, 0.1°, and 0.1° (Table 3) under the condition of flexion (FX), in the three models, respectively. Compared with 4.2° in the intact model, the changes in the ROM at the L4/5 level in the four motion conditions were − 97.62, − 97.62%, and − 97.62%, respectively (Table 4).

Under the condition of extension, the ROMs at the L4/5 level were 0.1°, 0.1°, and 0.1° (Table 3) in the three models, respectively. Compared with 3.9° in the intact model, the changes in ROM at the L4/5 level in the four motion conditions were − 97.44, − 97.44%, and − 97.44%, respectively (Table 4).

Under the condition of lateral bending, the ROMs at the L4/5 level were 0.2°, 0.2°, and 0.2° (Table 3) in the three models, respectively. Compared with 8.4° in the intact model, the changes in ROM at the L4/5 level in the four motion conditions were − 98.21, − 98.21%, and − 98.21%, respectively (Table 4).

Under the condition of lateral rotation, the ROMs at the L4/5 level were 0.1°, 0.1°, and 0.1° (Table 3) in the three models, respectively. Compared with 3.7° in the intact model, the changes in ROM at the L4/5 level in the four motion conditions were − 97.30, − 97.30%, and − 97.30%, respectively (Table 4).

### Intradiscal pressure at adjacent levels

**Fig. 10:**
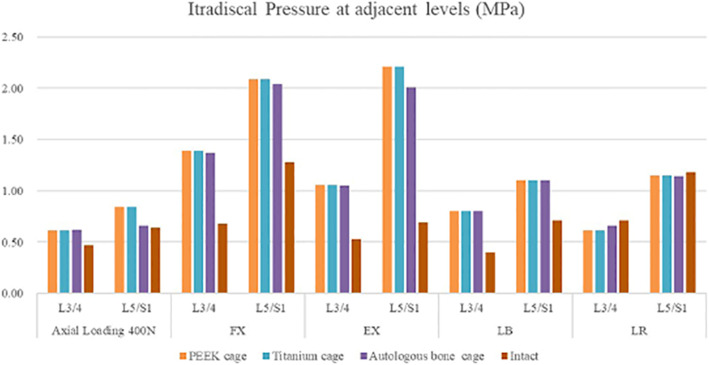
Intradiscal pressure at adjacent levels for various cages in 4 motion modes

**Fig. 11:**
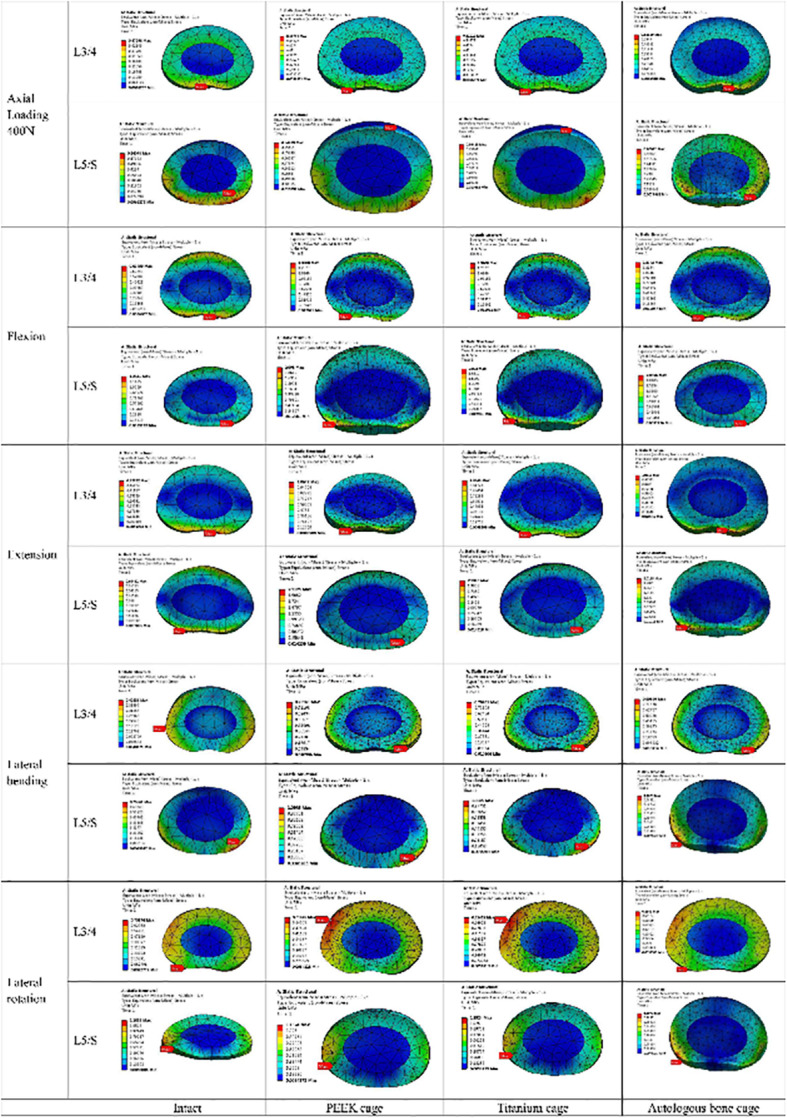
Cloud map of intradiscal pressure at adjacent levels for various cages in 4 motion modes

The intradiscal pressure (IDP) at adjacent levels are shown in Figs. 10 and 11 and Tables 5 and 6. Under all conditions, the IDP at the adjacent levels was calculated. The IDP at the adjacent levels was increased after conducting the treatment under axial loading of 400 N, flexion, extension, and lateral bending, but was decreased under lateral rotation. Notably, under axial loading of 400 N, flexion, extension, and lateral bending, the IDP at adjacent levels in the autologous bone-cage model increased less than that in the PEEK cage model and titanium cage model. Nevertheless, the IDP at the adjacent levels in the autologous bone-cage model was less increased compared with the other models.

**Table 5:** Intradiscal pressure at adjacent levels (MPa)

		PEEK cage	Titanium cage	Autologous bone cage	Intact
Axial Load 400 N	L3/4	0.61	0.61	0.62	0.47
	L5/S	0.84	0.84	0.66	0.64
FX	L3/4	1.39	1.39	1.37	0.68
	L5/S	2.09	2.09	2.04	1.28
EX	L3/4	1.06	1.06	1.05	0.53
	L5/S	2.21	2.21	2.01	0.69
LB	L3/4	0.80	0.80	0.80	0.40
	L5/S	1.10	1.10	1.10	0.71
LR	L3/4	0.61	0.61	0.66	0.71
	L5/S	1.15	1.15	1.14	1.18

**Table 6:** Change of intradiscal pressure at adjacent levels

		PEEK cage	Titanium cage	Autologous bone cage
Axial Load 400 N	L3/4	29.79%	29.79%	31.91%
	L5/S1	31.25%	31.25%	3.13%
FX	L3/4	104.41%	104.41%	101.47%
	L5/S1	63.28%	63.28%	59.38%
EX	L3/4	100.00%	100.00%	98.11%
	L5/S1	220.29%	220.29%	191.30%
LB	L3/4	100.00%	100.00%	100.00%
	L5/S1	54.93%	54.93%	54.93%
LR	L3/4	−14.08%	−14.08%	−7.04%
	L5/S1	−2.54%	−2.54%	−3.39%

### Facet joints force at adjacent levels

**Fig. 12:**
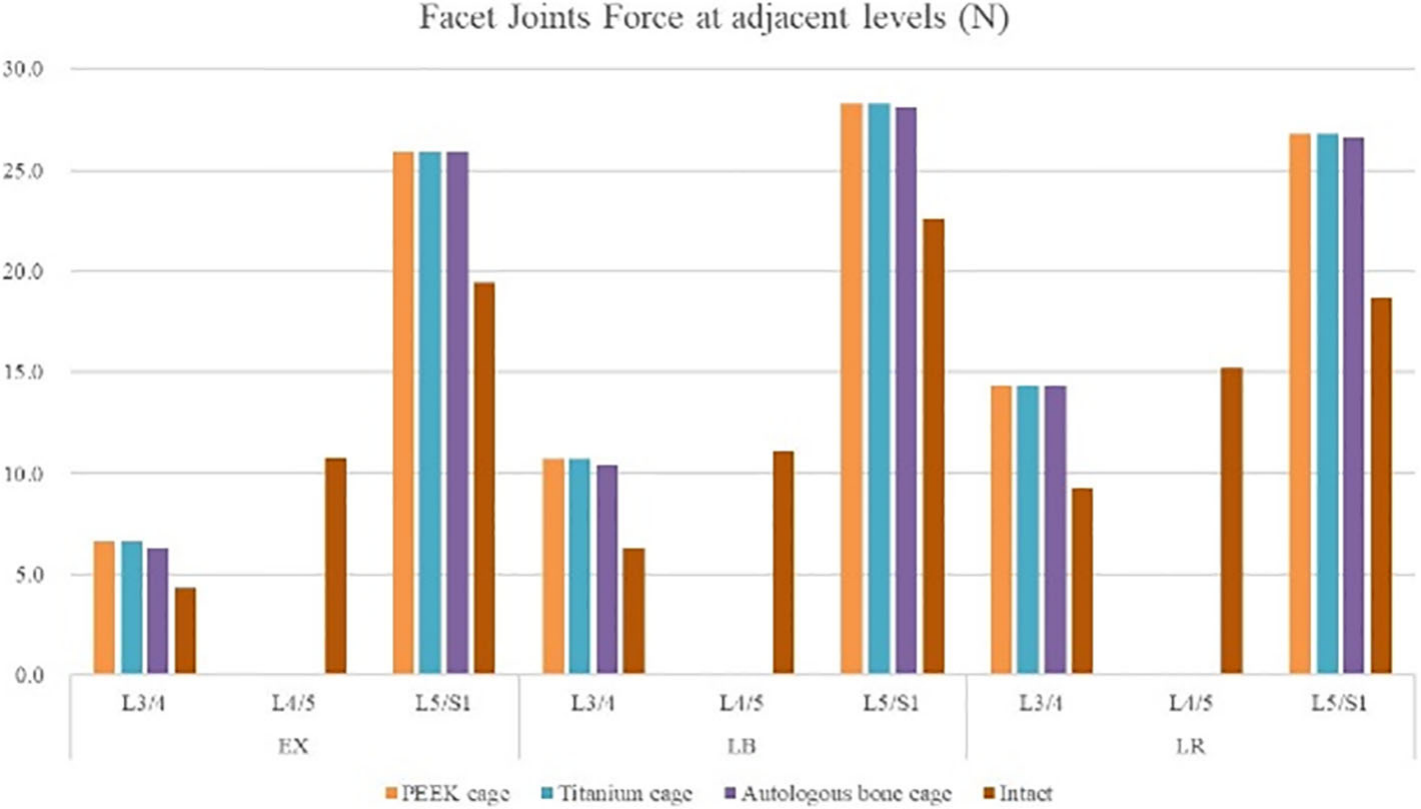
Facet joints force at adjacent levels for various cages in 4 motion modes

Due to the fixation at the L4/5 level, the facet joints force at L4/5 was not considered in this study. Considering that the facet joints would separate during flexion, facet joints force inflection was not considered as well. Under all these conditions, the facet joints force was shown to increase in all models (Fig. 12 and Table 7).

**Table 7:** Facet joints force at adjacent levels (N)

		PEEK cage	Titanium cage	Autologous bone cage
EX	L3/4	6.6	6.6	6.3
	L5/S	25.9	25.9	25.9
LB	L3/4	10.7	10.7	10.4
	L5/S	28.3	28.3	28.1
LR	L3/4	14.3	14.3	14.3
	L5/S	26.8	26.8	26.6

### The maximum stress in the endplate-cage interface at the treatment level

**Fig. 13:**
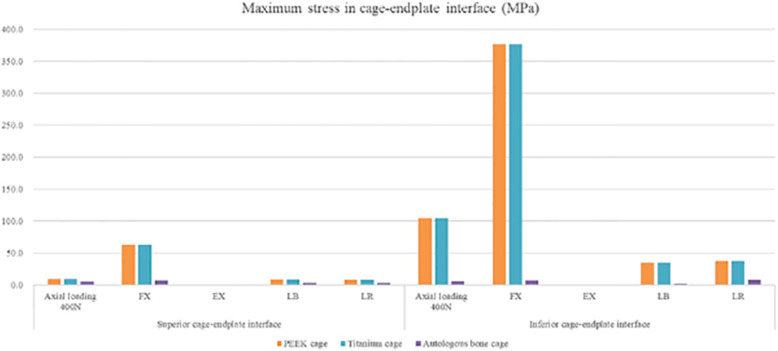
Maximum stress in the cage-endplate interface at surgical levels for various cages in 4 motion modes

**Fig. 14:**
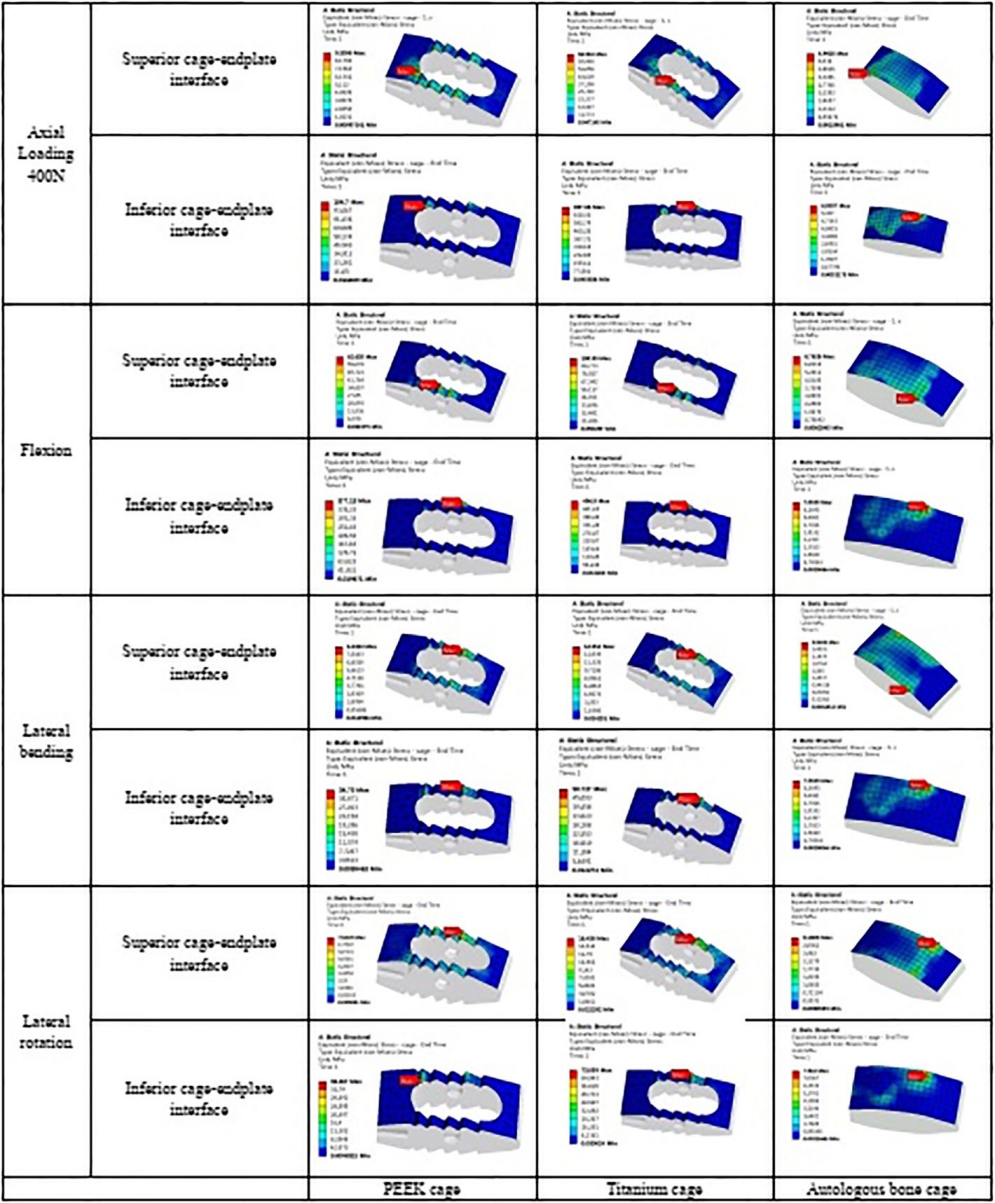
Cloud map of stress in the superior cage-endplate interface at surgical levels for various cages in 4 motion modes

Because of the separation of the cage and endplate during extension, the stress in the superior cage-endplate interface in extension was not considered. In the autologous bone-cage model, the maximum stress in the superior cage-endplate interface under all these conditions was much lower than that in other models. The maximum stress in the inferior cage-endplate interface was close to that in all the other models under extension, while the stress was much lower in the autologous bone-cage model under other conditions (Figs. 13 and 14, and Table 8).

**Table 8:** Maximum stress in cage-endplate interface (MPa)

		PEEK cage	Titanium cage	Autologous bone cage
Superior cage-endplate interface	Axial load 400 N	9.2	9.2	5.0
	FX	62.6	62.6	6.8
	EX	–	–	–
	LB	8.5	8.5	2.8
	LR	7.5	7.5	3.2
Inferior cage-endplate interface	Axial load 400 N	104.7	104.7	6.1
	FX	377.1	377.1	7.1
	EX	0.1	0.1	0.1
	LB	34.7	34.7	2.3
	LR	36.9	36.9	7.9

## Discussion

PLIF with posterior instrumented supplements has been used in the management of lumbar conditions for many years [3, 4]. With decades of development, neural injury, broad dissection of bone and muscle, and blood loss have been improved [3, 4].

The addition of autologous bone graft made from the iliac crest has been considered as the “gold standard”. The high fusion rate is achieved because of the provision of cellular factors that are favorable for bone healing, and the inherent properties of osteoconduction and osteoinduction [9, 16]. Nevertheless, the complications and morbidities such as infection, bleeding, pain at the donor site, and iliac fracture cannot be ignored [9, 17, 18]. Commonly, the spinous process and laminae are dissected during PLIF, so the use of autologous bone graft from the spinous process and laminae can avoid the complications and morbidities while using a bone graft from the iliac crest [6, 8, 9].

Cages made of titanium have been used in interbody fusion for many years. Although the fusion rate is high when using a titanium cage, a high risk of subsidence has been reported due to its high stiffness [1–5, 19]. PEEK has a stiffness close to that of cortical bone and has been widely used to produce cages [10, 20]. Furthermore, using PEEK cages in interbody fusion contributes to a nominal immediate anterior load sharing and restoration of height in the collapsed intervertebral space caused by degenerated discs [21]. The subsidence rate in the interbody fusion using PEEK cages was lower than that when using the titanium cages [2, 7, 22]. Nevertheless, the fusion rate was lower when using the PEEK cages because of its disadvantages of osteoconduction and osteoinduction [2, 7, 23].

To prevent complications when using autologous bone graft harvested from the iliac crest and to overcome the disadvantages of titanium or PEEK cages, a cage made from autologous bone was designed, and its biomechanical performance was evaluated using FEA. The biomechanical behaviors in various surgical models with three kinds of cages on ROM, the maximum stress in the cage-endplate interface, FJF, and IDP in four motion modes were evaluated. As shown in Fig. 9, Table 3, and Table 4, compared to the intact model, the ROM decreased by 97.30–98.21% at the surgical levels, while increased by 5.56–16.25% at adjacent levels in all surgical models, which was consistent with the results of the previous studies [11–14]. No significant differences were found in the ROM of the three surgical models, suggesting that the use of autologous bone-cage in PLIF could provide stability close to that of traditional solid cages. The reduced ROM at the surgical levels could be good for fusion and healing. As shown in Figs. 10 and 11, and Tables 5 and 6, the IDP at the adjacent levels increased in the surgical models during various motion modes, except for lateral rotation. The FJF at the L4/5 level disappeared in the surgical models because of the cages and rigid fixation with screws and rods. At the adjacent levels, the FJF increased in all motion modes in all surgical models. Nevertheless, no significant differences were found in FJF at the adjacent levels between the models. As shown in Figs. 13 and 14, and Table 6, the maximum stress in the cage-endplate interface was significantly higher in the surgical models of titanium cage and PEEK cage than that of the autologous bone-cage in all motion modes, except in extension. In the inferior cage-endplate interface in axial loading and flexion, the maximum stress in the surgical models with a titanium cage and PEEK cage were dozens of times higher than that in the surgical model with autologous bone-cage. Grant et al. measured the stiffness of different regions on the endplate and found a trend of decrease from the outside to the center of the endplate [24]. If the local stress was higher than the limit of the related regions, microfracture would occur [3, 4, 16–18, 24], leading to osteolysis and cage subsidence [3, 4, 16–18]. The stress in the cage-endplate interface was only less than 10 MPa in the autologous bone-cage model, which suggested that the lower the stress in the cage-endplate interface, the lower possibility of occurrence of microfractures, osteolysis or cage subsidence.

As the material property of the autologous bone-cage is unclear, the properties were set at a Young’s modulus of 5000 MPa, with a Poisson’s ratio of 0.29. The basis for setting the parameters was that the mixture of the spinous process and laminar bone obtained by compression is clinically roughly similar to that of cortical bone and cancellous bone in a 1:1 ratio. In the present study, the bone-cage after autogenous bone compression was considered as a whole cage. Therefore, no interaction was considered among the compressed parts. Nevertheless, it might influence the results, but further studies will be necessary to characterize the parts. This could provide some theoretical basis on what is happening, but clinically, it might have little value since the bone-cage made from compressed resected bones will be different from one patient to another. Especially, autograft tissue from spinous processes/lamina often contain soft tissue fragments that may change the biomechanical properties of the bone-cage, and this is a limitation of the model. The compressed cage may interfere with or even destroy the microstructure of bone cells. Due to the possible loss of growth factors in bone after extrusion, the time required for intervertebral fusion during our clinical case follow-up was found to be slightly longer than for the other commercial cages, but there was no difference in the overall fusion rates [8]. Some bone resorption could occur, but it did not affect the fusion rate in the clinical study [8]. In the near future, we will conduct further experiments to check whether the osteogenic performance of the compressed cage formed after extrusion has changed.

The limitations of our study should not be ignored. We compared parameters in our study with that of the obtained parameters from the model in the literature. The validation should be conducted by comparing the results in the FE study with that of biomechanical experiments and clinical studies. The FE model of the lumbar spine was simplified to improve the efficiency of convergence in the FE study. It still cannot represent the actual conditions in a real human body. Furthermore, the material property of the autologous bone-cage that was set in this study was still not the precise value, so more biomechanical studies should be conducted in the future.

## Conclusions

In conclusion, the results of the FEA showed that an interbody cage made from autologous bone could affect the biomechanical behavior noticeably in PLIF. Compared to the surgical models of a titanium cage and PEEK cage, the autologous bone-cage achieved to maintain the stability in lumbar interbody fusion. The autologous cage might have some advantages in stress in the cage-endplate interface, which could, in turn, decrease the subsidence rate. In addition, using autologous bone-cage in lumbar interbody fusion might achieve better fusion rate and healing in clinical practice. Nevertheless, more in vitro biomechanical experiments should be conducted to obtain the precise material property of autologous bone-cage. Further clinical studies are necessary to validate the effect of autologous bone-cages on stability, subsidence, clinical effect, and the results of this FEA study.

## Data Availability

The datasets used and/or analysed during the current study are available from the corresponding author on reasonable request.

## Abbreviations

CT: Computed tomography
FEA: Finite element analysis
FJF: Facet joint force
NURBS: Nonuniform rational b-spline surfaces
PLIF: Posterior lumbar interbody fusion
PEEK: Polyetheretherketone
ROM: Range of motion

## References

[1] DiPaola CP, Molinari RW (2008). Posterior lumbar interbody fusion. J Am Acad Orthop Surg.

[2] Nemoto O, Asazuma T, Yato Y, Imabayashi H, Yasuoka H, Fujikawa A (2014). Comparison of fusion rates following transforaminal lumbar interbody fusion using polyetheretherketone cages or titanium cages with transpedicular instrumentation. Eur Spine J.

[3] Wang H, Lv B (2018). Comparison of clinical and radiographic results between posterior pedicle-based dynamic stabilization and posterior lumbar intervertebral fusion for lumbar degenerative disease: a 2-year retrospective study. World Neurosurg.

[4] Schlegel KF, Pon A. The biomechanics of posterior lumbar interbody fusion (PLIF) in spondylolisthesis. Clin Orthop Relat Res. 1985;(193):115–19. 3971610

[5] Fogel GR, Parikh RD, Ryu SI, Turner AW (2014). Biomechanics of lateral lumbar interbody fusion constructs with lateral and posterior plate fixation: laboratory investigation. J Neurosurg Spine.

[6] Lin B, Yu H, Chen Z, Huang Z, Zhang W (2016). Comparison of the PEEK cage and an autologous cage made from the lumbar spinous process and laminae in posterior lumbar interbody fusion. BMC Musculoskelet Disord.

[7] Le TV, Baaj AA, Dakwar E, Burkett CJ, Murray G, Smith DA (2012). Subsidence of polyetheretherketone intervertebral cages in minimally invasive lateral retroperitoneal transpsoas lumbar interbody fusion. Spine (Phila Pa 1976).

[8] Hu MW, Liu ZL, Zhou Y, Shu Y, Chen CL, Yuan X (2012). Posterior lumbar interbody fusion using spinous process and laminae. J Bone Joint Surg Br.

[9] Wang L, Malone KT, Huang H, Zhang Z, Zhang Z, Zhang L (2014). Biomechanical evaluation of a novel autogenous bone interbody fusion cage for posterior lumbar interbody fusion in a cadaveric model. Spine (Phila Pa 1976).

[10] Vadapalli S, Sairyo K, Goel VK, Robon M, Biyani A, Khandha A (2006). Biomechanical rationale for using polyetheretherketone (PEEK) spacers for lumbar interbody fusion-A finite element study. Spine (Phila Pa 1976).

[11] Zhang Z, Li H, Fogel GR, Liao Z, Li Y, Liu W (2018). Biomechanical analysis of porous additive manufactured cages for lateral lumbar Interbody fusion: a finite element analysis. World Neurosurg..

[12] Zhang Z, Fogel GR, Liao Z, Sun Y, Liu W (2018). Biomechanical analysis of lumbar interbody fusion cages with various lordotic angles: a finite element study. Comput Methods Biomech Biomed Engin.

[13] Burkhart TA, Andrews DM, Dunning CE (2013). Finite element modeling mesh quality, energy balance and validation methods: a review with recommendations associated with the modeling of bone tissue. J Biomech.

[14] Zhong ZC, Wei SH, Wang JP, Feng CK, Chen CS, Yu CH (2006). Finite element analysis of the lumbar spine with a new cage using a topology optimization method. Med Eng Phys.

[15] Noailly J, Wilke HJ, Planell JA, Lacroix D (2007). How does the geometry affect the internal biomechanics of a lumbar spine bi-segment finite element model? Consequences on the validation process. J Biomech.

[16] Jenis LG, Banco RJ, Kwon B (2006). A prospective study of autologous growth factors (AGF) in lumbar interbody fusion. Spine J.

[17] Goulet JA, Senunas LE, DeSilva GL, Greenfield ML. Autogenous iliac crest bone graft. Complications and functional assessment. Clin Orthop Relat Res. 1997;(339):76–81. 10.1097/00003086-199706000-00011 9186204

[18] Heary RF, Schlenk RP, Sacchieri TA, Barone D, Brotea C (2002). Persistent iliac crest donor site pain: independent outcome assessment. Neurosurgery..

[19] Chen Y, Wang X, Lu X, Yang L, Yang H, Yuan W (2013). Comparison of titanium and polyetheretherketone (PEEK) cages in the surgical treatment of multilevel cervical spondylotic myelopathy: a prospective, randomized, control study with over 7-year follow-up. Eur Spine J.

[20] Kim YH, Choi DK, Kim K (2012). Investigation of the compressive stiffness of spinal cages in various experimental conditions based on finite element analysis. Proc Inst Mech Eng H.

[21] Abdul QR, Qayum MS, Saradhi MV, Panigrahi MK, Sreedhar V (2011). Clinico-radiological profile of indirect neural decompression using cage or auto graft as interbody construct in posterior lumbar interbody fusion in spondylolisthesis: which is better?. J Craniovertebr Junction Spine.

[22] Kim MC, Chung HT, Cho JL, Kim DJ, Chung NS (2013). Subsidence of polyetheretherketone cage after minimally invasive transforaminal lumbar interbody fusion. J Spinal Disord Tech.

[23] Olivares-Navarrete R, Gittens RA, Schneider JM, Hyzy SL, Haithcock DA, Ullrich PF (2012). Osteoblasts exhibit a more differentiated phenotype and increased bone morphogenetic protein production on titanium alloy substrates than on poly-ether-ether-ketone. Spine J.

[24] Grant JP, Oxland TR, Dvorak MF (2001). Mapping the structural properties of the lumbosacral vertebral endplates. Spine (Phila Pa 1976).

